# Protein Conformational Changes in the Bacteriorhodopsin Photocycle: Comparison of Findings from Electron and X-Ray Crystallographic Analyses

**DOI:** 10.1371/journal.pone.0005769

**Published:** 2009-06-02

**Authors:** Teruhisa Hirai, Sriram Subramaniam

**Affiliations:** Laboratory of Cell Biology, National Cancer Institute, National Institutes of Health, Bethesda, Maryland, United States of America; Illinois Institute of Technology, United States of America

## Abstract

Light-driven conformational changes in the membrane protein bacteriorhodopsin have been studied extensively using X-ray and electron crystallography, resulting in the deposition of >30 sets of coordinates describing structural changes at various stages of proton transport. Using projection difference Fourier maps, we show that coordinates reported by different groups for the same photocycle intermediates vary considerably in the extent and nature of conformational changes. The different structures reported for the same intermediate cannot be reconciled in terms of differing extents of change on a single conformational trajectory. New measurements of image phases obtained by cryo-electron microscopy of the D96G/F171C/F219L triple mutant provide independent validation for the description of the large protein conformational change derived at 3.2 Å resolution by electron crystallography of 2D crystals, but do not support atomic models for light-driven conformational changes derived using X-ray crystallography of 3D crystals. Our findings suggest that independent determination of phase information from 2D crystals can be an important tool for testing the accuracy of atomic models for membrane protein conformational changes.

## Introduction

**Figure 1:**
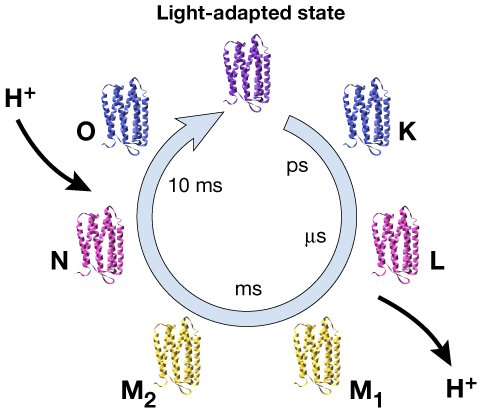
Photocycle of bacteriorhodopsin. Different intermediates formed in the photocycle are indicated with timescale of occurrence, sequence and approximate color corresponding to their spectroscopic signatures.

Bacteriorhodopsin (bR), a 27-kD membrane protein, functions as a light-driven proton pump in the membranes of the halophilic organism *H. salinarum*. A single molecule of retinal that is covalently attached to the protein via a protonated Schiff base serves as the chromophore for light absorption. Upon illumination, the Schiff base releases a proton to the extracellular medium, and is subsequently reprotonated from the cytoplasmic medium. The transmembrane proton gradient generated is available to drive other cellular functions such as transport of other ions or small molecules, synthesis of ATP, or rotation of the flagellar motor for cell motility. Following the pioneering electron crystallographic studies of Henderson, Glaeser and colleagues [1], [2] an atomic model for bacteriorhodopsin was first determined from cryo-electron microscopy by Henderson *et al* and confirmed and extended by work in a number of laboratories using the same method [3]–[6]. Extensive spectroscopic studies have provided a description of the sequence of intermediates generated during the photocycle (Fig. 1) [7], [8], while functional analysis of a large number of mutants [9]–[11] had identified key residues that line the path of the proton at different stages of transport.

The existence of large-scale light-driven protein conformational change in bacteriorhodopsin was first reported from analyses of projection maps at ∼7 Å resolution using both neutron [12] and X-ray [13], [14] diffraction patterns obtained from oriented membrane stacks. This initial work was extended by electron crystallographic studies [15]–[17] aimed at a detailed investigation of conformational changes during the photocycle by structural analysis of two-dimensional bacteriorhodopsin crystals trapped at various times after illumination, and under conditions that result in the selective accumulation of each of the late intermediates in the photocycle (see Fig. 1 for key intermediates in photocycle). From these experiments it was proposed that in wild-type bacteriorhodopsin a single, large protein conformational change in the cytoplasmic region occurs within ∼1 ms after illumination, approximately coincident with deprotonation of the Schiff base. This change persists at least through the N intermediate of the photocycle, perhaps with small variations, and is reversed upon the thermal regeneration of the starting protein conformation. Subramaniam *et al* proposed the simplified view that the overall structures of the initial state and the early intermediates (K, L, and M_1_) are approximated by one protein conformation (cytoplasmically closed form), while the structures of the late intermediates (M_2_, N) are well approximated by the other protein conformation, (cytoplasmically open form)[17].

A number of atomic models describing this conformational change have been reported over the years using both electron and X-ray crystallographic studies [18]–[22]. Most X-ray studies were carried out by analyzing the structure of bacteriorhodopsin in three-dimensional crystals following illumination, while the electron crystallographic studies were performed by analyzing the structure of the D96G/F171C/F219L triple mutant, which displays the full extent of the conformational change in the absence of illumination. Both electron and X-ray crystallographic studies of the three-dimensional structure of the cytoplasmically open state used the known structure of the ground state as a starting point for the refinement, without including phase information from the conformationally altered state. In this study, direct electron microscopic imaging of two-dimensional crystals of the D96G/F171C/F219L triple mutant was used to derive phase information to ∼7 Å in projection. By combining the phase data with amplitude data obtained from electron diffraction patterns, we produced vector difference maps [23], which were subsequently compared with vector difference maps calculated using phase and amplitude data from coordinates deposited in the Protein Data Bank (PDB). Comparison of our findings with those from recent X-ray crystallographic studies show that while the X-ray analyses using three-dimensional crystals have the potential to provide a higher resolution description of regions that are not involved in light-induced structural changes, the reported structures do not reflect the large conformational change observed in electron crystallographic studies with two-dimensional crystals.

## Results

### Comparative analysis of deposited coordinates for bacteriorhodopsin

**Figure 2:**
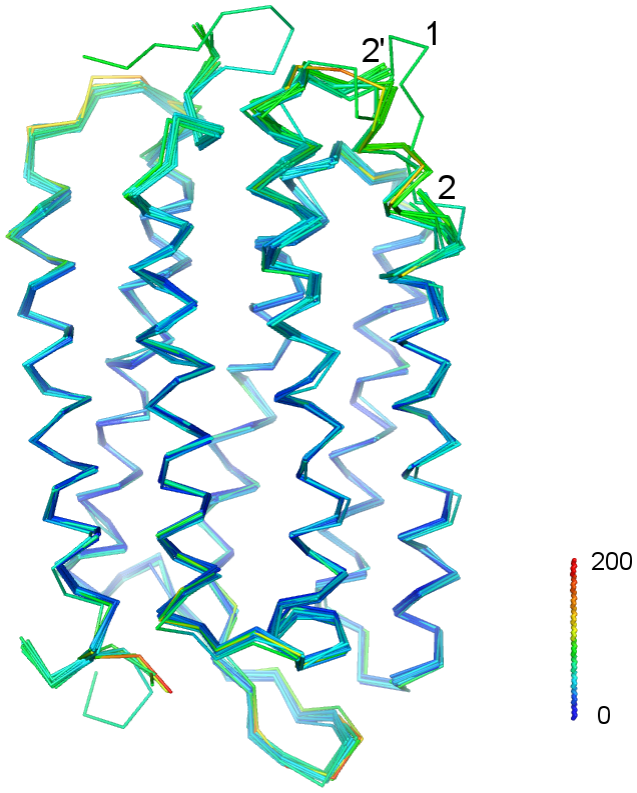
Ribbon representation of all ground state and cytoplasmically closed state. All ground state and cytoplasmically closed state (K, L and M_1_) listed in Table 1 are used. All coordinates are aligned with 1M0L using the LSQMAN program [51]. Models are colored based on the B-factors of that coordinate set using color spectrum provided by Pymol (Color bar is shown in the right corner.). 1CWQ(A) shows a unique shape of the loop around loop EF (“1”).

To evaluate the similarities and differences among reported structures for bacteriorhodopsin, the coordinates reflecting the unilluminated state and the early intermediates (until the M_1_ state; Table 1) were aligned (see Materials and methods for a detailed description; Fig. 2). Models were colored based on the temperature factors for each coordinate set using the color spectrum (blue to red from low to high B-factor) provided by Pymol (http://pymol.sourceforge.net/). Overall, the various coordinates for the unilluminated state and the early intermediates are fairly well-aligned, with fluctuations primarily around the loop regions. Among the loops, the EF loop (residues 157–161) and the contiguous section of helix E (residues 153–156) show the greatest variability. These regions also have high temperature factors (Fig. 2). One set of coordinates for the unilluminated state, 1CWQ(A) [24], shows systematic and significant deviations (marked “1” in Fig. 2) from all other sets of coordinates. These deviations are largely localized to the regions displaying the highest temperature factors. Another smaller, but systematic, deviation is observed in the cytoplasmic ends of helices E and F between the sets of coordinates that include the EF loop region and those that have left this region out of the model (marked 2 and 2′, respectively, in Fig. 2). It is unclear whether this difference indicates the start of a conformational change around the missing EF loop region or an artifact arising from incomplete constraints due to the missing residues in the model.

**Table 1:** bR coordinates used in figures.

PDB code (chain or model ID)	Sample	Crystallization method	State, illumination, pH condition	Occupancy (%)	Resolution (Å)	Residues in the model	Reference
1QHJ	WT	lipidic cubic phase	Ground		1.9	5–232	Belrhali *et al*, 1999[54]
1C3W	WT	lipidic cubic phase	Ground		1.55	5–156, 162–231	Luecke *et al*, 1999b[55]
1FBB	D96G/F171C/F219L	2D crystal/EM	Ground		3.2	4–227	Subramaniam & Henderson, 2000[27]
1KGB	WT	lipidic cubic phase	Ground		1.65	5–156, 162–231	Facciotti *et al*, 2001[37]
1IW6	WT	membrane fusion	Ground		2.3	5–231	Matsui *et al*, 2002[30]
1M0L	WT	lipidic cubic phase	Ground		1.47	5–156, 162–231	Schobert *et al*, 2002[29]
1F50	E204Q	lipidic cubic phase	Ground		1.7	5–156, 162–231	Luecke *et al*, 2000[41]
1C8R	D96N	lipidic cubic phase	Ground		1.8	5–156, 162–231	Luecke *et al*, 1999a[42]
1QKP	WT	lipidic cubic phase	K, 110 K green	35	2.1	5–232	Edman *et al*, 1999[28]
1M0K(2)	WT	lipidic cubic phase	K, 100 K green	40	1.43	5–156, 162–231	Schobert *et al*, 2002[29]
(1)			Ground				
1IXF	WT	membrane fusion	K, X-ray	13	2.6	5–231	Matsui *et al*, 2002[30]
1E0P(B)	WT	lipidic cubic phase	L, 170 K green	70	2.1	5–232	Royant *et al*, 2000[34]
(A)			Ground				
1O0A (1)	WT	lipidic cubic phase	L, 170 K red	∼60	1.62	5–156, 162–231	Lanyi & Schobert, 2003[35]
(2)			Ground				
1UCQ	WT	membrane fusion	L, 160 K green, 100 K red	20	2.4	5–231	Kouyama *et al*, 2004[33]
1VJM(2)	WT	lipidic cubic phase	L, 150 K red	50	2.3	5–232	Edman *et al*, 2004[36]
(1)			Ground				
1KG8	WT	lipidic cubic phase	M_1_, 230 K yellow	100	2	5–155, 167–231	Facciotti *et al*, 2001[37]
1M0M(2)	WT	lipidic cubic phase	M_1_, 210 K red or yellow	60	1.43	5–156, 162–231	Lanyi & Schobert, 2002[38]
(1)			Ground				
1P8H(1)	WT	lipidic cubic phase	M_1_, 295 K yellow	42	1.52	5–156, 162–231	Schobert *et al*, 2003[39]
(2)			Ground				
1FBK	D96G/F171C/F219L	2D crystal/EM	M		3.2	4–228	Subramaniam & Henderson, 2000[27]
1CWQ(B)	WT	lipidic cubic phase	M, RT green	35	2.25	2–239	Sass *et al*, 2000[24]
(A)			Ground				
1IW9	WT	membrane fusion	M, X-ray	∼70	2.5	5–231	Takeda *et al*, 2004[40]
1F4Z	E204Q	lipidic cubic phase	M_2_, RT yellow	>93	1.8	5–156, 162–231	Luecke *et al*, 2000[41]
1C8S	D96N	lipidic cubic phase	M_N_, 290 K yellow	100	2	5–153, 176–222	Luecke *et al*, 1999a[42]
1P8U(1)	V49A	lipidic cubic phase	N′, 295 K red	37	1.62	5–156, 162–231	Schobert *et al*, 2003[39]
(2)			Ground				
1JV7	D85S	lipidic cubic phase	O		2.25	9–63, 78–232	Rouhani *et al*, 2001[25]
1X0I	WT	membrane fusion	O, acidic blue		2.3	8–233	Okumura *et al*, 2005[26]

**Figure 3:**
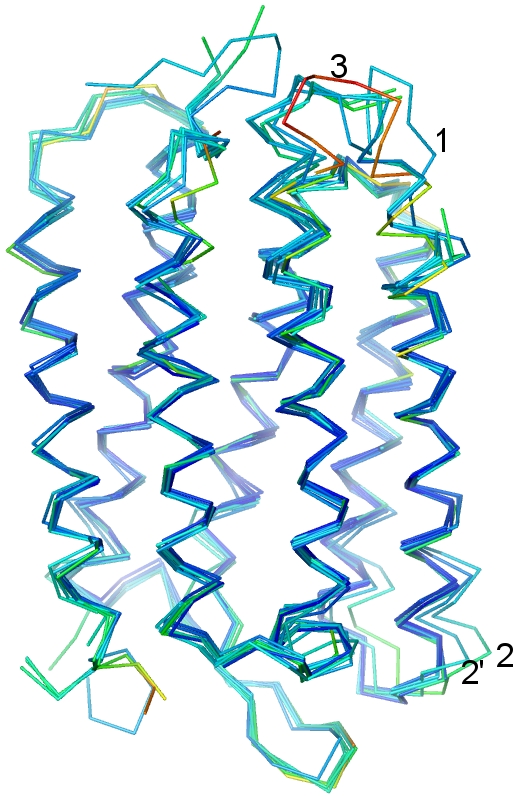
Ribbon representation of all cytoplasmically open and later state (M_2_, N′ and O). All cytoplasmically open and later state (M_2_, N′ and O) listed in Table 1 are used. All coordinates are aligned with 1M0L in the same way as in Fig. 2. Eight coordinates (1CWQ(B), 1IW9, 1F4Z, 1C8S, 1P8U(1), 1JV7, 1X0I and 1FBK) are used. 1CWQ(B) displays a unique loop structure around loop EF (“1”). 1JV7 and 1X0I show a unique conformational change around loop DE (“2” and “2′”). 1FBK shows a larger movement in the EF loop region (“3”). Color scheme for B-factor is the same as in Fig. 2.

All M_2_ and later state structures (eight coordinate sets: 1CWQ(B), 1IW9, 1F4Z, 1C8S, 1P8U(1), 1JV7, 1X0I and 1FBK), which are expected to contain larger deviations from the unilluminated state, were aligned using 1M0L as a reference, and are shown in Fig. 3 with the same coloring according to temperature factor described in Fig. 2. Of this group, three sets of coordinate sets (1CWQ(B), 1JV7 and 1X0I) are different from the others. 1CWQ(B) includes distinct loop structures around the EF loop (“1”), which, as pointed out above, are already apparent in the ground state coordinates 1CWQ(A)[24]. 1JV7[25] and 1X0I[26], proposed O intermediate analogues, display a unique conformational change consistent with an unusually large outward deviation in the DE loop at the extracellular side (marked “2” and “2′” in Fig. 3). The remaining five structures show good agreement amongst themselves and with the transmembrane regions of 1CWQ(B), except in the region around loop EF and the cytoplasmic ends of helices E, F, and G. Interestingly, in the set of late intermediate structures, the deviations around the EF loop are larger than those seen among the structures of the unilluminated state, consistent with the proposed location of the large protein conformational change in bacteriorhodopsin upon illumination [27]. The coordinates derived by electron crystallographic analysis of the late intermediate analog 1FBK show the largest movement of loop EF (marked “3” in Fig. 3).

**Figure 4:**
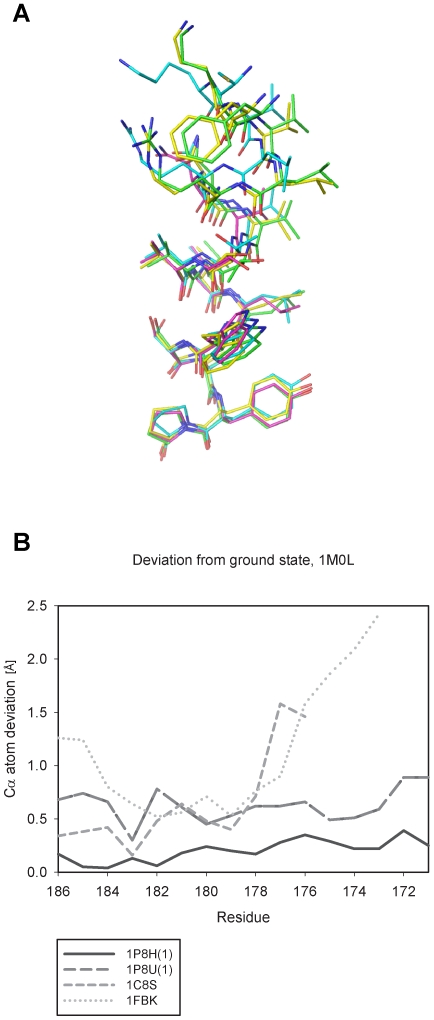
Superposition and Cα atom deviation of the helix F region from M_1_ to N′ intermediates. (A) Superposition of the helix F region from M_1_ to N′ intermediates. Residues 171–186 of 1P8H(1), 1P8U(1), 1C8S, and 1FBK are extracted and displayed as “lines” modeled in Pymol. Residue number increases from top (cytoplasmic side) to bottom (periplasmic side) showing larger deviations around the cytoplasmic side. (B) Cα atom deviation of M_1_ to N′ states from ground state coordinates, 1M0L at each atom position in helix F. 1C8S lacks the cytoplasmic side of helix F.

To investigate the conformational differences among the various sets of coordinates in the regions where the largest changes occur, we superimposed the regions containing residues 171–186 of helix F from the structures reported as models for the M_1_ to N′ states (1P8H(1), 1P8U(1), 1C8S, and 1FBK) (Fig. 4A). A plot of the extent of the variation is presented in Fig. 4B using the coordinates for the unilluminated state, 1M0L, as a reference. The level of variation observed among these structures is progressively higher towards the cytoplasmic end of the helix (up in Fig. 4A and right in Fig. 4B). The largest displacement corresponds to the 1FBK set of coordinates, which was derived from electron crystallographic analysis. Only 1C8S and 1FBK show significant and progressive trends in structural change towards the cytoplasmic end of helix F. Residues 171–175, where the largest deviations are observed in 1FBK, are not modeled in 1C8S, but there is excellent correspondence in the magnitude of the deviation in the region (residues 176–179) for which coordinates have been deposited in PDB for both 1C8S and 1FBK. These analyses led us to conclude that although all coordinate sets intended to provide models for the late intermediate states of bacteriorhodopsin show some level of variation in the cytoplasmic end of helix F with respect to the unilluminated state, only 1C8S and 1FBK show systematic and similar trends in the extent of the conformational change consistent with tilting of helix F in the photocycle. The other sets of coordinates (1P8H(1), 1P8U(1)), generate models that are generally not consistent with this structural change.

**Figure 5:**
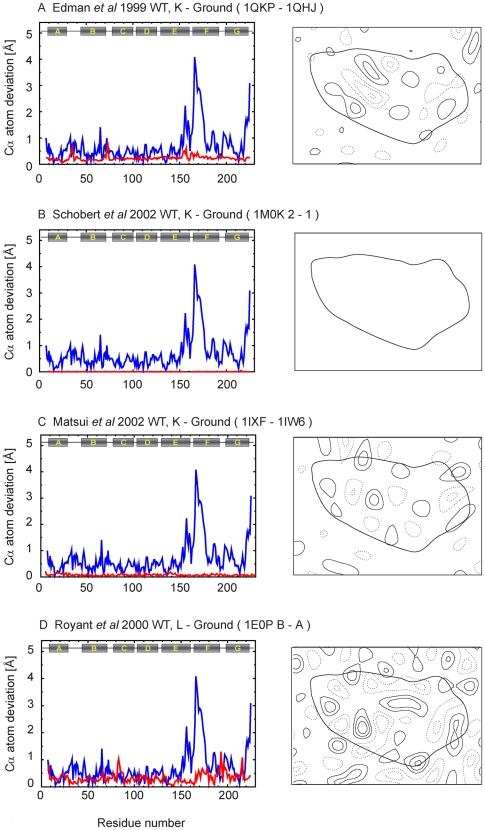
Cα atom deviation plots and difference maps between intermediate and ground states calculated at 7 Å. In the Cα atom deviation plots, red line shows the Cα atom deviation between intermediate and ground state after alignment using LSQMAN. For some plots, there are one or two gaps because there are missing residues either for intermediate or ground states. Blue line shows the deviation between 1FBK and 1FBB as a measure of reference. To calculate difference maps, we used common residues between intermediate and ground state; the retinal was always included. Lipids and water molecules were always excluded.

To complement the analysis in Fig. 4B that compares the extent of the conformational change, we calculated for each of the coordinates pairwise Cα atom deviations, as well as difference maps for each of the intermediates relative to the corresponding unilluminated states, from coordinates deposited in the PDB database (Table 1). A complete set of these plots and projection maps is presented in Figs. 5–9. All difference maps are calculated at 7-Å resolution to clearly show helix movements, with a contour level defined proportionally to the sigma value of the corresponding ground state map density.

**Figure 6 pone-0005769-g006:**
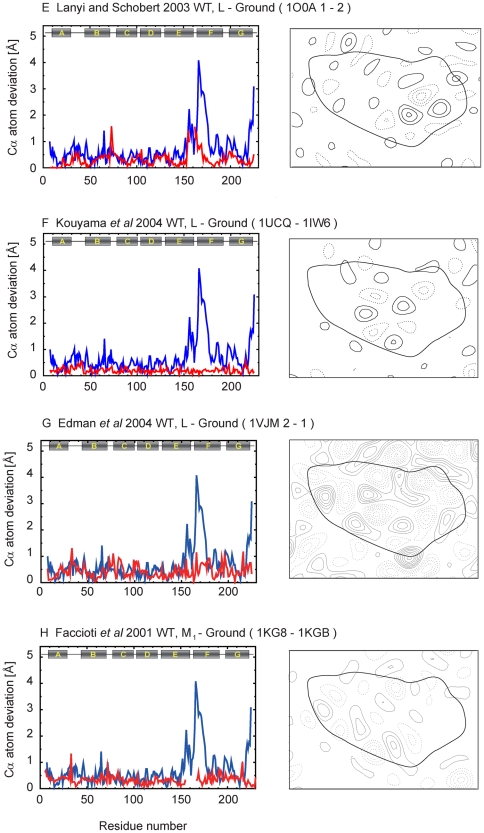
Cα atom deviation plots and difference maps between intermediate and ground states calculated at 7 Å (continued).

**Figure 7 pone-0005769-g007:**
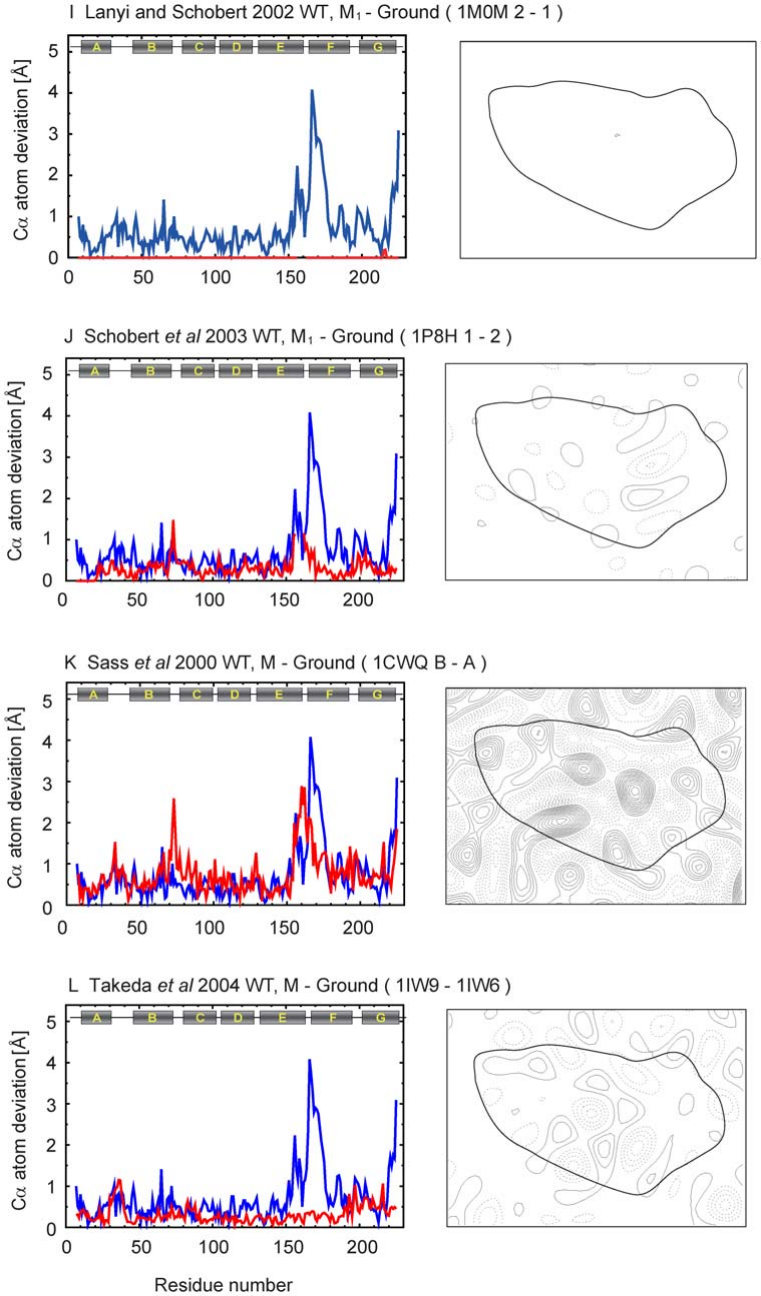
Cα atom deviation plots and difference maps between intermediate and ground states calculated at 7 Å (continued).

**Figure 8 pone-0005769-g008:**
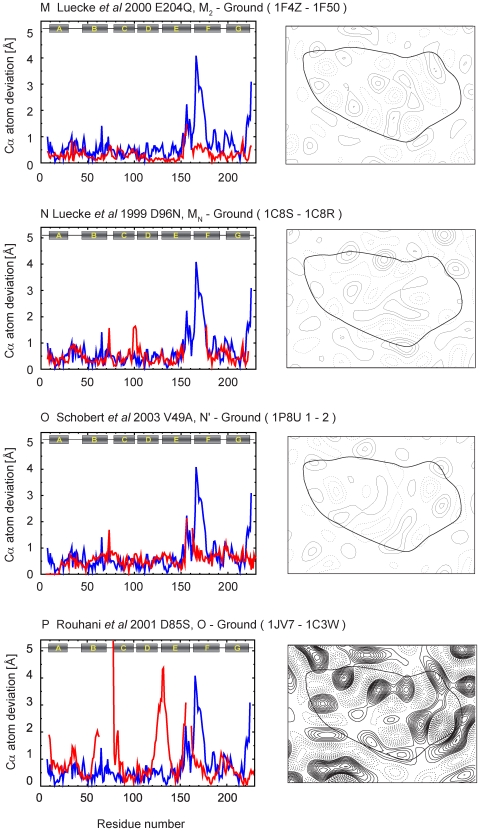
Cα atom deviation plots and difference maps between intermediate and ground states calculated at 7 Å (continued).

**Figure 9:**
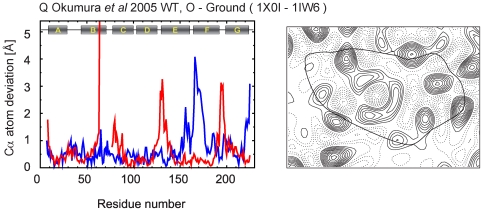
Cα atom deviation plots and difference maps between intermediate and ground states calculated at 7 Å (continued).

The K and L intermediates appear early in the photocycle, within ∼1 ps and ∼1 ms, respectively, after illumination at room temperature. For the K intermediate, the conformational changes with respect to the ground state are limited mainly to the retinal and its vicinity and are expected to result in no significant helix displacements. This expectation is confirmed in the difference maps derived from all three sets of coordinates reported for the K intermediate state (Figs. 5A–C) [28]–[30]. In the case of the L intermediate, there are discrepancies among results from different groups. To date, three groups have reported a total of four L intermediate structures [31], [32]. One shows minimal structural changes (Fig. 6F) [33], while the others show discernible structural changes at the level examined here (Figs. 5D, 6E, and G) [34]–[36]. The nature of the structural changes reported in the latter models is, however, different. In the structure for the L intermediate deposited by Edman *et al* (Fig. 6G)[36], as reported also in a preceding model by the same group (Fig. 5D) [34], there is a local bend of helix C toward the central channel, with an outward movement of helix F, in contrast to the results by Lanyi and Schobert (Fig. 6E)[35], which do not include these helical displacements.

In the M_1_ intermediate, the Schiff base is deprotonated, likely without the extensive protein conformational changes that begin with the subsequent formation of the M_2_ intermediate. Three different sets of coordinates have been reported so far for the M_1_ intermediate state (Figs. 6H, 7I, and J)[37]–[39]. Two of these sets report the same kind of structural changes for the M_1_ intermediate trapped following illumination at 230 K and 210 K. These changes are minimal in one set (Fig. 7I) [38] and greater in the other (Fig. 6H) [37], especially in the vicinity of helix F. The third set (Fig. 7J)[39] reports results obtained from accumulation of the M_1_ intermediate at 295 K, and in this case the structural changes are closer to those seen by the same group for the L intermediate (Fig. 6E) [35]. In summary, results from different groups agree that no significant conformational changes of the helices occur in the K state, but they diverge with regard to the nature and extent of such structural changes in the L and M_1_ intermediate stages.

The discrepancies among the numerous studies of the late intermediate states by crystallographic analysis of three-dimensional crystals of wild-type and mutant bacteriorhodopsin proteins, illuminated under conditions aimed to accumulate M, M_2_, M_N_, N′ intermediates, are even greater. Sass *et al* illuminated lipidic cubic phase crystals of wild-type bacteriorhodopsin at room temperature to capture the M intermediate, and reported a refinement in which they assumed that only a third of the molecules in the crystal are converted. Despite a relatively low crystallographic resolution (2.25 Å), low occupancy of the intermediate, and high level of twinning in their crystals, Sass *et al* reported a shift of helix F in the region from W189 to W182 by about 0.8 Å towards the cytoplasmic side and an outward tilt in the region from V179 to V167, with the largest bending from L174 to N176 (Fig. 7K)[24]. Takeda *et al* presented a different structure for the M intermediate, using crystals of wild-type bacteriorhodopsin prepared by the membrane fusion method, from a higher occupancy (∼70%). Their structure for M includes significant displacements of helix G, the AB loop, and the cytoplasmic end of helix B but no appreciable changes in helices A, D, E, and F (Fig. 7L)[40].

Lipidic cubic phase crystals of the E204Q mutant (Fig. 8M)[41] and the D96N mutant (Fig. 8N)[42] illuminated at room temperature have been used to observe the M_2_ and M_N_ intermediates of the photocycle, respectively. In both, the occupancy of the M states was nearly 100%. In the E204Q mutant, release of the proton to the extracellular side occurs late in the photocycle with decay of the M_2_ intermediate, which results in accumulation of this intermediate. In the case of the D96N mutant, reprotonation of the Schiff base is greatly slowed, resulting in accumulation of what has been referred to as the M_N_ intermediate. The extent of structural changes reported for these two closely related M intermediates is small, but nevertheless they are very different as seen in the projection maps (Figs. 8M and N).

Studies on the N′ and O intermediates using selected mutants have also shown disparate results. Schobert *et al* used the V49A mutant to observe the N′ intermediate with illumination at room temperature at pH 5.6. The N′ state is formed after reprotonation of the Schiff base by Asp96 and the subsequent reprotonation of Asp96 from the cytoplasmic bulk. In its reported structure, at a relatively high resolution (1.63 Å); the principal changes are vertical displacements of helices F and G without significant tilts or rotation (Fig. 8O) [39]. Both models related to the O intermediate (D85S mutant and acidic blue form), reported by Rouhani *et al* (Fig. 8P)[25] and Okumura *et al* (Fig. 9Q)[26], have significant and unique movements of the channel on the extracellular side (Fig. 3).

### Conformational changes confirmed by electron crystallography using experimental phase information

Electron crystallography has been used in parallel with X-ray studies to study the conformational changes in bacteriorhodopsin after illumination. The projection maps of intermediate stages obtained with electron crystallography [17] revealed the large conformational change that occurs in late intermediates. The three-dimensional map of the open state analog, the D96G/F171C/F219L triple mutant, showed a large movement of helices E and F with respect to the ground state [27]. These previous projection maps were built with amplitude data from diffraction patterns collected from either intermediate or wild type bacteriorhodopsin, but with phase data from wild type bacteriorhodopsin only. To build the atomic model of the triple mutant (1FBK), amplitude data derived directly from experimental diffraction patterns of the mutant was used [27] in a strategy similar to that used for molecular replacement techniques used in X-ray crystallography. Since phase information can be directly obtained from images of the mutant, we used this in the present work to obtain more accurate difference maps. Details of how we processed the triple mutant images are described in the Materials and methods section.

**Figure 10:**
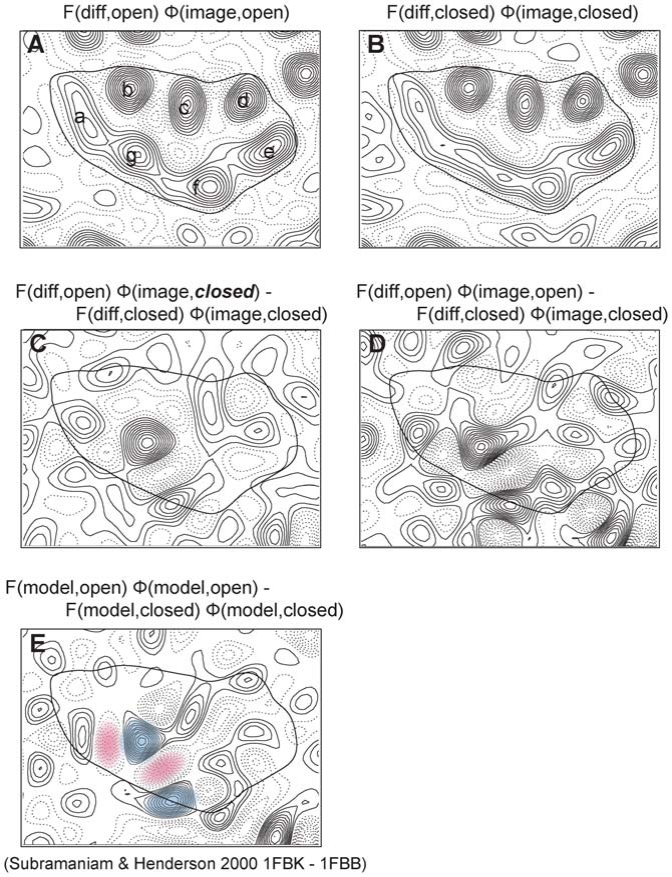
Maps calculated using EM data at 7 Å. Amplitude data for closed and open states from diffraction analysis alone (F_(diff, open)_, F_(diff, closed)_) were obtained as described in the text. These amplitude data were used to build models for 1FBB and 1FBK [27]. Phase data for closed state (φ_(image, closed)_) are the same as those of Henderson *et al* [3]. Phase data for open state (φ_(image, open)_ ) were calculated as described in the text. (A) Projection map of open state, F_(diff, open)_ φ_(image, open)_. Amplitude data is from diffraction patterns and phase data is from images. (B) Projection map of closed state, F_(diff, closed)_ φ_(image, closed)_. Amplitude data is from diffraction patterns and phase data is from images. (C) Difference map, F_(model, open)_ φ_(model, *closed*)_ - F_(model, closed)_ φ_(model, closed)_. Phase information comes only from the model of the closed (i.e. unilluminated) state. The absence of phase information is a common situation when molecular replacement is used in X-ray or electron crystallography. (D) Difference map, F_(diff, open)_ φ_(image, open)_ - F_(diff, closed)_ φ_(image, closed)_. For both open and closed states, amplitude data is from diffraction patterns and phase data is from images of each state. (E) Difference map, F_(model, open)_ φ_(model, open)_ - F_(model, closed)_ φ_(model, closed)_. All amplitude and phase data are calculated from coordinates 1FBK and 1FBB. The pair of positive (blue) and negative (magenta) peaks correspond to the movements of the cytoplasmic end of helix F, the EF loop and cytoplasmic end of helix G.

The projection maps for the open and closed states of bacteriorhodopsin shown in Figs 10A and B were calculated at 7-Å resolution using a data combination with phase information from the images and amplitude information from diffraction patterns. To confirm the conformational change between the open and closed states several projection difference maps were calculated (Figs 10C–E). For the map calculated in Fig. 10C, only amplitude data from the open state (triple mutant) was used, unlike the map shown in Fig. 10D. The absence of experimental phase information is a common scenario in X-ray and electron crystallography when molecular replacement techniques are used to determine structures of proteins using only diffraction data in combination with the previously known structure of a closely related protein. Projection difference maps reported for bacteriorhodopsin have also been calculated using this strategy in previous neutron, X-ray and electron crystallographic analyses [17]. Despite the inherent shortcomings in this approach, the maps bear a strong resemblance to the vector difference maps shown in Figs 10D and E. Even though the use of amplitude data from the open state provides enough information to detect the large protein conformational change, the combination of both amplitude and phase data from open state gives the most accurate difference map (Fig. 10D). This difference map is very similar to the difference map (Fig. 10E) previously derived using 1FBK and 1FBB [27] theoretical models, especially around the regions supporting the main movements. The pair of positive (blue) and negative (magenta) peaks those correspond to the main movements in Fig. 10E are also found in Fig. 10D. The pattern around helices E–F is less similar compared with other helices and that might come from the high temperature factor around the E–F loop. Difference maps Fig. 10C and D were compared with Fig. 10E in terms of phase residual values. The phase residual between Fig. 10D and E was 29.0° in the range of 15 to 10 Å (27 peaks) while Fig. 10C and E gave 60.1° (90° random) as phase residual over the same range. The excellent consistency between Fig. 10D, the projection difference map calculated from the best combination of experimental data and Fig. 10E, the projection difference map calculated from models supports the movements of helices F and G obtained by electron crystallographic analysis. As shown in Figs. 5–9, only the 1FBK set of coordinates derived by electron crystallography shows a calculated difference map that matches the experimentally observed map.

### Structural distribution of cytoplasmically open-state coordinates

**Figure 11:**
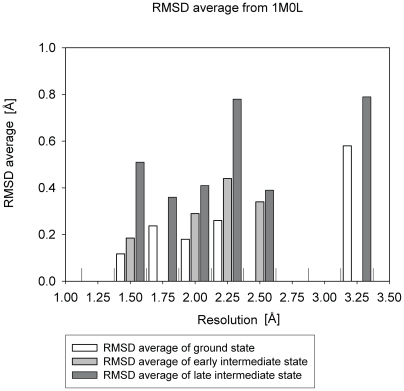
RMSD average of ground, early, and late intermediate state coordinates from 1M0L ground state coordinates. The height of each vertical bar represents the average value of RMSD in that resolution range (0.25 Å interval). 10 early intermediate and 6 late intermediate coordinates (Table 1) are used for each calculation. 15 ground state coordinates (Table 1) are used for calculation of ground state. All RMSD values used for this figure are listed in Fig. S1. Furthermore, structural distribution of late intermediate coordinates is discussed in Fig. S2.

As previously discussed [43], the structures of bacteriorhodopsin reported by X-ray crystallography at a given resolution show a resolution-dependent deviation from the averaged (ideal) structure. To discuss the conformational change between the intermediate and ground states of bacteriorhodopsin, it is necessary to take into account this type of structural ambiguity. As expected, the root mean square deviation (RMSD) average of ground and early and late intermediate state coordinates from the 1M0L ground state coordinates [29] (see Materials and methods section) correlated positively with resolution (Fig. 11). Early intermediate state coordinates showed a slightly larger difference from the 1M0L than ground state coordinates. For these cases, it would be difficult to distinguish real conformational changes from structural uncertainty even if there were a change. The late intermediate state structure appears following the M_1_ state with opening of the cytoplasmic half channel. Six structures (1FBK, 1CWQ(B), 1IW9, 1F4Z, 1C8S, and 1P8U) of late intermediates were used to calculate the RMSD average shown in Fig. 11. Unlike early intermediate coordinates, these show significantly larger RMSD from the 1M0L coordinates compared with ground state coordinates on average. In particular, the RMSD average from the 1M0L coordinates is high at the resolution range of 2.25 Å because of the high RMSD value of the late intermediate coordinates 1CWQ(B) with respect to the ground state 1M0L. The coordinates 1CWQ(B) have unique profile as explained in Figs 3 and 5K. The RMSD average for late intermediate states is also high at the resolution range of 1.5 Å as the structure of the 1P8U(1) set of coordinates seems to show a greater deviation than what would be expected based on the nominal resolution.

## Discussion

In this study, we compare available data from different reports on the structures of intermediate stages of bacteriorhodopsin. The comparison reveals that the structures for the same intermediate reported by multiple research groups are not only significantly different, but are also not on the same trajectory of conformational change of the protein. We believe that the steric constraints resulting from crystal packing may be responsible in part for these discrepancies which are discussed in greater detail in this section.

As is widely accepted in the field, light-driven proton pumping must involve the participation of a cytoplasmically open state that accepts protons from the cytoplasmic medium to pump them to the outside. X-ray analyses from oriented membrane stacks [14], [44]–[46], electron paramagnetic resonance (EPR) experiments [47], and several electron microscopic studies [17] have suggested that the movements of helices F and G are involved in such an opening of the cytoplasmic region. The same kind of movement was reported for the N intermediate state after studying an N-intermediate analog, the F219L mutant trapped after illumination, by electron microscopy of tilted 2-dimensional crystals [48]. The cytoplasmically closed state of unilluminated bacteriorhodopsin has a tightly packed hydrophobic region that includes the location of the F219. The rationale for using the F219L mutant was that Phe219 has been replaced with a smaller residue (Leu), which could make the cytoplasmically closed state less stable. This allows the N intermediate of the F219L mutant to accumulate in contrast to the wild-type photocycle [49].

We carefully revisited the processing of diffraction data and the refinement process of the model (Figs. S3, S4, S5) to ensure the accuracy of these processes and the modeling of 1FBK. In addition, the new difference map presented in this study with direct experimental phase information (Fig. 10D) produced without any model bias confirms the fidelity of the 1FBK model. It is possible, however, that the mutant structure does not exactly represent the cytoplasmically open state that occurs during the photocycle. The D96G/F171C/F219L triple mutant was designed as an extension of the F219L mutant. Replacing these three amino acids (Asp96, Phe171, and Phe219) in the tightly packed area of the cytoplasmically closed state with smaller residues favors the cytoplasmically open conformation, even in the absence of illumination. The three modified residues are located around the cytoplasmic half of the channel and these locations do not establish any tight contacts in the open state of either the wild-type or the triple mutant protein. Thus, those regions responsible for the open-state conformation remain intact. The structure of 1FBK was solved at a modest resolution (3.2 Å) and we found that the loop region has a high temperature factor compared with other X-ray structures (Fig. 3). Even if the EF loop is flexible to some extent, the agreement between the new experimental projection map and the projection map from the models indicates that the 1FBK is a good model for the structure of the EF loop.

In this paper, our discussion focuses on the large-scale conformational change found among the reported bacteriorhodopsin structures. In addition to this large conformational change, there are many small inconsistencies (retinal conformation, orientation of the side chain, position of the water molecule and the hydrogen bond, etc.) among the structures reported for several intermediate stages, including K and L [31], [32], which can be important when discussing the mechanism of proton pumping. These minor inconsistencies might be due to several causes. The three-dimensional crystals made by the lipidic cubic-phase method (P6_3_ crystal packing) and the fused two-dimensional crystals (p3 crystal packing) usually have twinning and require careful analysis. Even under optimal conditions for illumination, several intermediate states could coexist in the illuminated samples. Thus, it may be difficult to distinguish the contribution of a given intermediate structure from that of other molecules in the mixtures, especially if the occupancy of this intermediate is not high and the resolution is limited. In addition, other conditions, such as actual pH, might affect the structure locally. Radiation damage can also be responsible for decarboxylation of aspartate and glutamate residues, loss of the hydroxyl group from tyrosines and the methylthio group from methionines, as discussed by Matsui *et al* [30]. If the resolution is not high enough, especially in regions with high temperature factor, the resulting structure will be uncertain. In some reports, uncertain areas have not been included in the final coordinates reported, while others performed modeling within the allowable uncertainty to derive plausible structures. As a consequence, there is a wide variation in protein and side chain conformation in the weaker and more disordered regions of the map; this situation can change with improved order in the crystals and resolution of the data.

What is the reason that different analyses of the late intermediates differ significantly from each other? Can the reasons listed above to explain the inconsistencies among structures also explain this larger discrepancy? There is a good agreement among the ground state structures at the large scale reported by different groups. Also, the structures of the backbone of the K and L intermediates are similar. If, for example, X-ray radiation damage were an issue regarding the consistency of the backbone for intermediate structures on a large scale, the problem should have arisen also for the structures of the ground state. The inconsistency among structures of later state intermediates might be due to differences in the preparation of the intermediate sample as well as in processing the data from the ground state. If the estimation of occupancy is not right, the model will be refined against data resulting from the mixture of different conformations, which will affect the large-scale conformation, especially if the sample contains both cytoplasmically closed and open states. The fact that some cytoplasmically closed structures exhibit the characteristics of open-state conformation may be related to this issue.

Two distinct differences exist between the experimental design that led to the 1FBK model and the other experiments discussed here. First, electron crystallography was used to determine the 1FBK coordinates while X-ray crystallography was used to determine the other coordinate sets. Second, the model was determined from the D96G/F171C/F219L triple mutant instead of a photointermediate of wild-type bacteriorhodopsin. Most likely, both factors are important because both are relevant to the steric hindrance of the conformational change in the crystal lattice. The large conformational changes in the photocycle might not be easily accommodated for three-dimensional crystals, as discussed by Lanyi and Schobert [50]. Difference maps for the D96N mutant calculated from X-ray and electron diffraction using two-dimensional crystals were reported previously [13], [17] and those projection maps show the same movement that we confirmed with the triple mutant. Nevertheless, the question of why the large-scale movement observed in the two-dimensional crystals has not yet been reported in three-dimensional crystals is relevant. Crystals of the D96N mutant were grown in the lipidic cubic phase and the P6_3_ packing has exactly the same packing structure as the two-dimensional crystals (p3) in the horizontal plane but with additional contact between planes. It is possible that the crystal forces in the three-dimensional crystal inhibit any significant rearrangement of helices that would normally occur upon illumination, and re-crystallization in the new conformation may be required. When the structure of 1C8S was determined, increases in temperature factors were reported in the regions where we have observed the largest conformational changes, and those regions were excluded from the model of 1C8S. On the other hand, as has been discussed above, the remainder of 1C8S is in good agreement with the 1FBK model. This suggests that the conformational change that can occur in the two-dimensional crystals is incomplete in three-dimensional crystals. Taken together, we conclude that the three-dimensional crystals used in X-ray crystallographic analysis of light-driven conformational changes in bacteriorhodopsin prevent observation of physiologically relevant protein conformational changes that occur with each cycle of proton transport, and can be observed by electron crystallography of two-dimensional crystals. The ability to experimentally determine image phases from cryo-electron microscopy of 2D crystals is thus a powerful tool that can be used for validation of conformational changes in membrane proteins derived by either electron or X-ray crystallography.

## Materials and Methods

### Structural alignment and calculation of RMSD and difference maps

To align coordinates for comparison and to calculate RMSD values between coordinates, the helical regions (9–29 (helix A), 43–62 (helix B), 82–94 (helix C), 108–128 (helix D), 134–152 (helix E), 178–191 (helix F), and 205–221 (helix G)) were used with the LSQMAN software [51]. To calculate RMSD average from the 1M0L ground state coordinates, 10 early intermediate and 8 late intermediate coordinates were used for each calculation (Table 1). The 16 ground state coordinate sets that correspond to those intermediates were used for calculation of the ground state. Cα atom deviations and difference maps were calculated between the intermediate and corresponding ground states. 17 coordinates pairs were used and the pair of 1FBK and 1FBB was used as a reference. The CCP4 package [52] was used to calculate and contour the maps from those data. The difference maps were calculated at 7-Å resolution with a contour level defined proportionally to the sigma value of the corresponding ground state map density. Contour lines were drawn every 10% of the sigma value; dotted line indicates negative contour. Before calculating difference maps, ground state structure was aligned with 1FBB using LSQMAN and both ground state and corresponding intermediate state were aligned to the same frame of reference to obtain difference maps in the same view.

### Preparation of specimens

Two-dimensional crystals of wild-type bacteriorhodopsin and the D96G/F171C/F219L triple mutant suitable for analysis by electron crystallographic were obtained as described [17]. In contrast to fused membranes obtained from wild-type bacteriorhodopsin and most other single mutants previously studied, the fused membranes obtained from the D96G/F171C/F219L triple mutant display a considerable amount of twinning. The presence of twinning necessitated the use of smaller crystalline areas of <2 µm^2^ (∼2×10^5^ molecules) for collecting diffraction patterns.

Typically, 3 µl of a suspension of two-dimensional crystals of the triple mutant was deposited on copper/rhodium grids coated with carbon (thickness ∼75–125 Å). After incubation for 30 seconds, most of the droplet was removed by “edge-on” blotting with filter paper. With the grid still wet, a 5-µl droplet of a sugar solution (either 1% glucose, 3% trehalose, or 5% trehalose) was deposited on the grid. After quick mixing of the sugar solution with the crystalline suspension, the grid was blotted fully, also by edge-on blotting. The speed with which the meniscus receded from the grid surface provided a useful indicator of the likely quality of the specimen. Flatter specimens were obtained when the meniscus receded cleanly without leaving a more slowly drying residual aqueous film. The grids were plunged immediately (within 5–10 seconds after blotting) into liquid nitrogen and transferred into the specimen chamber of a Gatan cold stage pre-cooled to liquid nitrogen temperatures.

### Processing of electron microscopic images

The images of the triple mutant used in this work were recorded using conventional low-dose procedures on a CM200 microscope (FEI/Philips Corporation, Oregon, USA) operating at 200 kV equipped with field emission gun at a magnification of 50,000× with defocus value around 21,000–25,000 Å. Images were scanned and digitized using a Zeiss SCAI scanner (Z/I Imaging Corporation, AL, USA) at 7-µm step. The size of well ordered areas used for image processing was about 6,000×6,000 pixels (70 µm^2^) on average. Three images were processed and merged using the MRC package [53]. The total number of reflections better than or equal to IQ4 used for merging was 115 up to 7 Å and 164 up to 3.4 Å. The number of unique reflections after merging was 31 up to 7 Å and 71 up to 3.4 Å. The *R*_merge_ was 30.6 up to 7 Å and 28.2 up to 3.4 Å. The overall phase residual was 17.7° up to 7 Å and 31.9° up to 3.4 Å. CCP4 package was used to calculate and contour the maps from those reflections.

## Supporting Information

Figure S1 RMSD data used for Fig. 11.
(0.09 MB PDF)

Figure S2 RMSD among late intermediate coordinates. In this figure we calculated the RMSD deviations among late intermediate structures for all possible combinations. RMSD of each possible combination between 6 late intermediate coordinates (M_2_∼N′, 6×5/2 = 15 cases) is plotted as a circle according to the lower resolution of that pair. Several circles that showed relatively smaller RMSD in that resolution range are colored in red or green and they are discussed later. For comparison RMSD average in each resolution range (0.25 Å interval) was shown as a vertical bar for ground state coordinates or late intermediate coordinates. RMSD values were calculated using the same region where 1M0L is modeled (residues 5–156, 162–231) to ensure that the comparisons were valid across all sets of coordinates. Some coordinate sets, like 1C8S, lack more residues than 1M0L and the RMSD was calculated using the set with the fewer residues. To account for this, the RMSD average of late intermediates from 1M0L was calculated in two ways. The first calculation was conducted simply using the region that overlaps with 1M0L (residues 5–156 and 162–231). The second calculation was conducted using the region where the 1C8S is modeled (residues 5–153 and 176–222) excluding a large area (residues 154–175); this second calculation is represented as 1M0L′ in the figure legend. In principle, the bar graphs of 1M0L′ showed a better RMSD than the bar graph of 1M0L because the latter includes less relatively higher temperature factor regions. Especially the resolution range of 3.25 Å where 1FBK belongs showed a large improvement because the cytoplasmic part of helices E, F, and G of 1FBK significantly deviate from 1M0L. We find that the RMSD distribution between the coordinates of different intermediates is higher than the average RMSD between ground state coordinates suggesting that not all intermediate coordinates represent the same structure. Some combinations have larger RMSD values even when compared to the average RMSD of intermediate coordinates from the ground state coordinates of 1M0L. This means that the movement of intermediate B (point “B”) from ground state (point “O”) is not in the same direction as that of intermediate A (point “A”), and intermediate B is not coming any closer to intermediate A starting from the ground state in structural space (ö^→^ *AB*ö≡ö^→^ *OB* - ^→^ *OA*ö > ö^→^ *OA*ö, ö^→^ *OB*ö). 1CWQ(B) tends to show a higher RMSD compared with other structures. 1P8U also appears to have a unique movement even though the movement is not as large as 1CWQ(B). Those comparisons that displayed equal or smaller RMSD than would be expected based on the differences which might arise only from resolution differences are colored in green or red. 1C8S seems to locate in the center of the open state structure distribution. The 1C8S, 1F4Z, and 1IW9 coordinate sets appear to form a cluster giving the better RMSD value among them (green circles). 1C8S also has a good RMSD value against 1FBK considering the resolution of 1FBK (red circle). The RMSD between 1F4Z or 1IW9 and 1FBK is slightly higher compared with either of the three against 1C8S. One of the reasons that 1C8S can belong to two clusters (1F4Z, 1IW9, and 1C8S or 1FBK and 1C8S) is that 1C8S is missing a large region around the EF loop (residues 154–175) and the cytoplasmic end of G (residues 223–228). 1F4Z, 1IW9, and 1C8S are coordinate sets which did not deviate much from the ground state (1M0L) as shown in Figs. 7L,8M, and N. The RMSD between these coordinates calculated using only the rigid helical part gave excellent agreement. 1FBK shows large conformational changes but these are located in the EF loop and in the cytoplasmic ends of helices F and G; these are exactly in correspondence to the missing parts in 1C8S (Fig. 4B). The RMSD average from 1M0L′ was usually better than RMSD between 1C8S and intermediates suggesting that the movement of 1C8S is not shared with other intermediates or not significant enough at this resolution for other intermediates. In other words, the core part of late intermediate structure is well represented by 1M0L than by 1C8S. As a summary, large movements found in 1CWQ(B) and 1FBK do not seem to be shared with any other structures. In those parts of the structure where coordinates exist for 1C8S, there is close similarity with other structures that correspond to the ground state of bacteriorhodopsin. In contrast, the model derived by electron crystallography (1FBK) shows significant and clear differences from ground state structure, mainly in the regions which are absent in the 1C8S coordinates.
(0.16 MB PDF)

Figure S3 Electron diffraction patterns used to build cytoplasmically open state model. Diffraction patterns were recorded at 120 kV using a CCD camera with 1150×1150 pixels as described before [56]. (a) 30° tilt. (b) 60° tilt. Here we present details of how the electron diffraction patterns were recorded from two-dimensional crystals embedded in a thin film of either glucose or trehalose for determination of the 1FBK structure reported in Subramaniam and Henderson (2000) [27]. The rationale for using sugars such as glucose or trehalose is that we presume they maintain the intrinsic order in the crystal by minimizing its contact with the surface of the carbon film. Untilted diffraction patterns could be recorded to better than 3-Å resolution from most specimens without much difficulty. However, it was considerably more difficult to reproducibly obtain good diffraction patterns at high specimen tilts. At higher tilts, the diffraction patterns are “blurred” in the direction perpendicular to the tilt axis. This is due to vertical distortions (i.e., lack of flatness) introduced in the crystal upon its contact with the carbon film. As a consequence, the angle between the electron beam and the normal to the plane of the crystal is not constant across the entire crystal, and the intensities of spots at higher z* values become spread out over progressively larger areas in the direction perpendicular to the tilt axis. This problem can be partially overcome by recording data from smaller areas of the crystal. Thus, for tilted specimens, diffraction patterns were collected from areas (∼1–1.5 µm^2^) which were small enough to be sufficiently flat, but large enough to provide a good signal/noise ratio for detection of weak and higher order reflections. There was no clear correlation observed between the qualities of patterns obtained from specimens prepared with either glucose or trehalose; however, it was often the case that in a given session, depending on the age and hydrophobicity of the carbon, the use of one or the other sugar resulted in a better specimen. A total of about 1000 diffraction patterns were included in the initial set, out of which 486 patterns were chosen by careful visual inspection for further processing. Criteria for selection were primarily based on minimal blurring of spots in the direction perpendicular to the tilt axis with the purpose of including data from only reasonably flat two-dimensional crystals.
(0.64 MB PDF)

Figure S4 Selected lattice line and the profile of merged set projected to the plain. (A) Selected lattice line, (2,8). (B) The profile of the fully merged set projected to the plain. Point spread function is also shown. Each diffraction pattern was automatically indexed, and the spot intensities were integrated using either a raster (for patterns recorded at specimen tilts less than 30°) or using profile fitting (for patterns recorded at specimen tilts greater than 30°). Each pattern was then compared to the curves recorded for wild-type bacteriorhodopsin in glucose at −100°C [57] and the relative proportions of the four different twins were determined. This exercise was performed with all four theoretically possible relative orientations of the crystal axes with those of the reference curves to ensure that the data were merged correctly. From the initial set of 486 patterns chosen, 286 minimally twinned diffraction patterns were selected in which the major twin proportion was greater than 0.8. These 286 patterns were merged using the wild-type lattice lines as a reference and lattice lines were fitted to the data to obtain an initial approximately merged set of lattice lines describing the structure of the triple mutant. The original set of 486 patterns was then merged against the new lattice curves to redetermine the twin proportions more accurately. The merging parameters for each crystal were inspected carefully again, and 84 crystals for which the major twin proportion was less than 0.70 were excluded from the data set. The remaining 402 untwined diffraction patterns were used to generate a new set of curves using the initial merge set of curves as a reference, and were self-merged to create a further improved set of lattice lines. The nearly merged data were further refined using an improved estimate of sigma values for each reflection, and by the inclusion of an individual weight factor for each diffraction pattern using procedures described by Grigorieff *et al* [4]. Two cycles of this refinement were carried out to obtain a final set of merged curves. The curves were sampled at 1/100 Å (approximately twice the thickness of the membrane) to obtain a set of intensities at H, K, and L values so that the data could be further processed with standard crystallographic programs.
(0.15 MB PDF)

Figure S5 Density (2F_O_ - F_C_) map of bR triple mutant and refined model (1FBK). In the initial stages of refinement for the previous model, a minimal starting model containing only the transmembrane regions of bacteriorhodopsin was used in a simplified least squares refinement with the PROLSQ program. Coordinates from 6 different starting models (2BRD, 1BRR, 1AP9, 1BRX, 1AT9, and 2AT9) were tested using diffraction data sets obtained both from wild-type bacteriorhodopsin and the triple mutant. We used the diffraction data for refinement of wild-type bacteriorhodopsin reported by Ceska and Henderson [57] as well as those used in the earlier electron crystallographic refinement reported by Grigorieff *et al* [4]. After a systematic and thorough evaluation, the 1BRR set of coordinates [58] was used as a starting model for the next stage of refinement using the CNS system [59], which involved simulated annealing followed by temperature factor refinement. The validity of the final map was tested by completely omitting from the starting model a series of test residues such as F42, W86, W189, and F208, or various combinations of residues at the cytoplasmic ends of helices F or G. In each case, the difference maps (F_O_-F_C_) obtained at the end of the refinement were unambiguous and clear density peaks were observed for each of the omitted regions.
(0.94 MB PDF)
