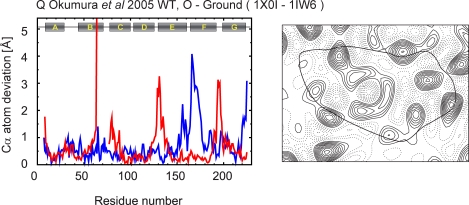# Correction: Protein Conformational Changes in the Bacteriorhodopsin Photocycle: Comparison of Findings from Electron and X-Ray Crystallographic Analyses

**DOI:** 10.1371/annotation/dab20871-1ee8-473c-92b0-2a4550b4b09a

**Published:** 2009-06-24

**Authors:** Teruhisa Hirai, Sriram Subramaniam

The published Figures 4, 5, 6, 7, 8, and 9 are illegible. Please see the corrected figures here:

Figure 4, 

**Figure:**
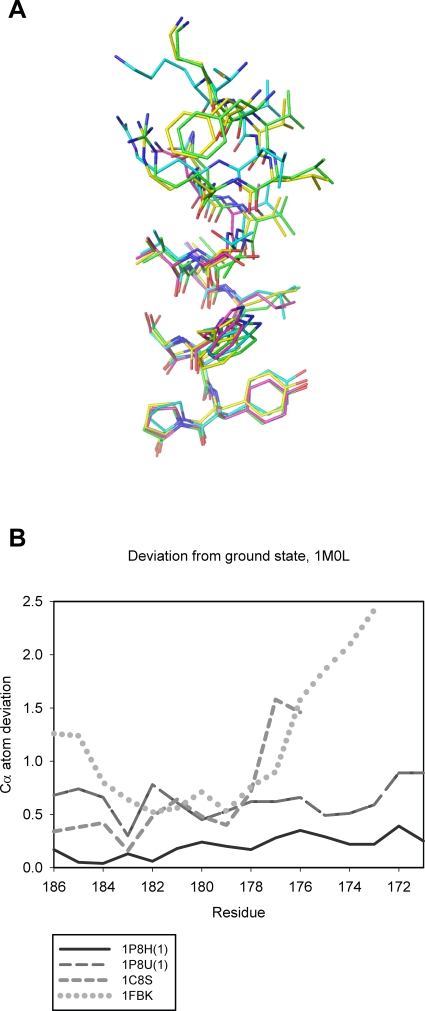


Figure 5, 

**Figure:**
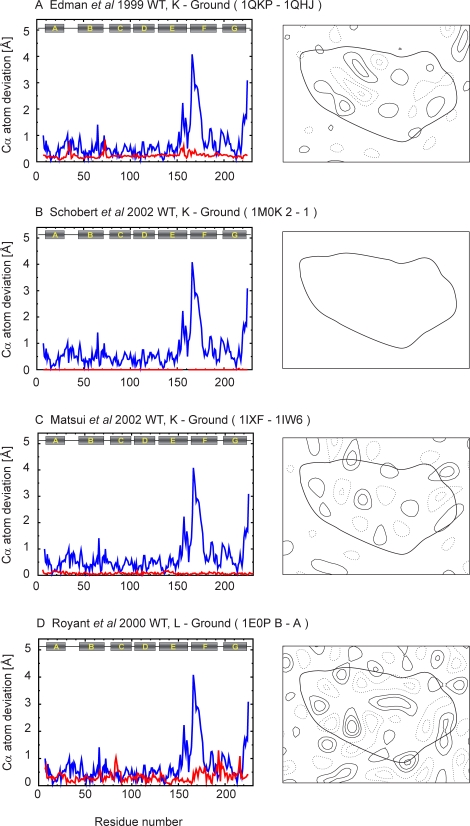


Figure 6, 

**Figure:**
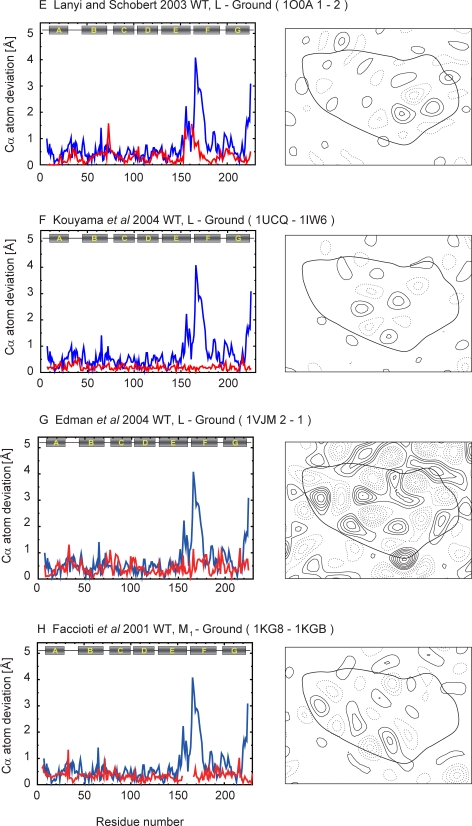


Figure 7, 

**Figure:**
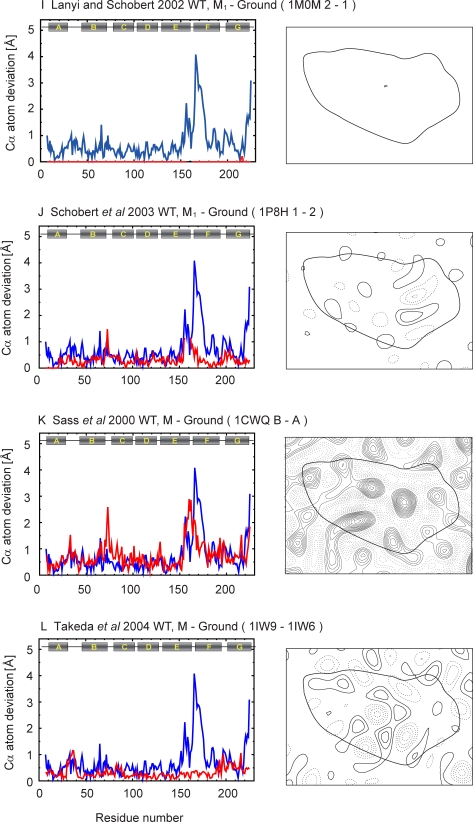


Figure 8, 

**Figure:**
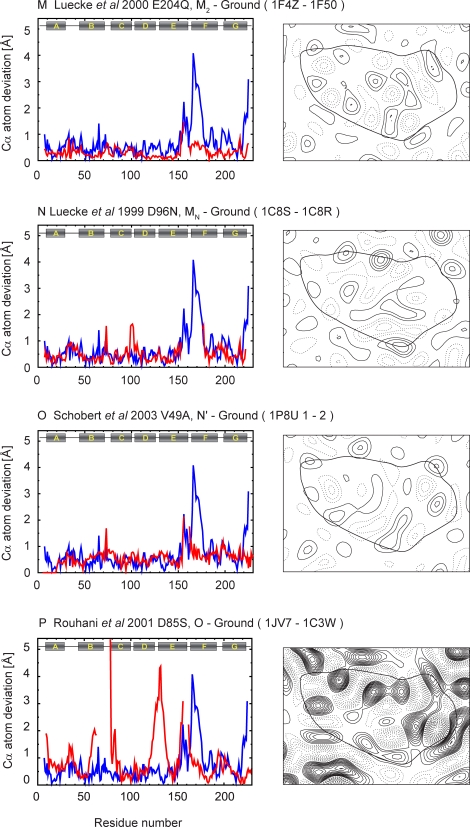


Figure 9, 

**Figure:**