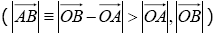# Correction: Protein Conformational Changes in the Bacteriorhodopsin Photocycle: Comparison of Findings from Electron and X-Ray Crystallographic Analyses

**DOI:** 10.1371/annotation/b7d18825-44b9-4ff1-9478-321049172908

**Published:** 2009-07-10

**Authors:** Teruhisa Hirai, Sriram Subramaniam

The equation in the fifteenth sentence of the legend for Figure S2 does not appear correctly. Please view the correct equation and related text here: 

This means that the movement of intermediate B (point “B”) from ground state (point “O”) is not in the same direction as that of intermediate A (point “A”), and intermediate B is not coming any closer to intermediate A starting from the ground state in structural space