# Supplementary material for: Protein Conformational Changes in the Bacteriorhodopsin Photocycle: Comparison of Findings from Electron and X-Ray Crystallographic Analyses
Source: PLoS One. 2009 Jun 2;4(6):e5769. doi: 10.1371/journal.pone.0005769 (PMC2685002; doi:10.1371/journal.pone.0005769)
Supplement: Figure S1 — RMSD data used for Fig. 11. (0.09 MB PDF) [file pone.0005769.s001.pdf]

Ground state

Resolution range (Å)	PDB code	Resolution	RMSD from 1M0L (Å)	Number of coordinate	Sum of RMSD (Å)	Average of RMSD (Å)
1.375 - 1.625	1M0K(1)	1.43	0.07	6	0.70	0.117
	1M0M(1)	1.43	0.17			
	1P8H(2)	1.52	0.03			
	1C3W	1.55	0.25			
	1P8U(2)	1.62	0.07			
	1O0A(2)	1.62	0.11			
1.625 - 1.875	1KGB	1.65	0.14	3	0.71	0.237
	1F50	1.70	0.32			
	1C8R	1.80	0.25			
1.875 - 2.125				2	0.36	0.180
	1QHJ	1.90	0.18			
	1E0P(A)	2.10	0.18			
2.125 - 2.375	1CWQ(A)	2.25	0.29	3	0.78	0.260
	1IW6	2.30	0.31			
	1VJM(1)	2.30	0.18			
2.375 - 2.625				0		
2.625 - 2.875				0		
2.875 - 3.125				0		
3.125 - 3.375	1FBB	3.20	0.58	1	0.58	0.580

Early intermediate

Resolution range (Å)	PDB code	Resolution	RMSD from 1M0L (Å)	Number of coordinate	Sum of RMSD (Å)	Average of RMSD (Å)
1.375 - 1.625	1M0K(2)	1.43	0.07	4	0.74	0.185
	1M0M(2)	1.43	0.18			
	1P8H(1)	1.52	0.26			
	1O0A(1)	1.62	0.23			
1.625 - 1.875				0		
1.875 - 2.125	1KG8	2.00	0.28	3	0.87	0.290
	1QKP	2.10	0.19			
	1E0P(B)	2.10	0.40			
2.125 - 2.375	1VJM(2)	2.30	0.44	1	0.44	0.440
2.375 - 2.625	1UCQ	2.40	0.36	2	0.68	0.340
	1IXF	2.60	0.32			
2.625 - 2.875				0		
2.875 - 3.125				0		
3.125 - 3.375				0		

Late intermediate

Resolution range (Å)	PDB code	Resolution	RMSD from 1M0L (Å)	Number of coordinate	Sum of RMSD (Å)	Average of RMSD (Å)
1.375 - 1.625	1P8U(1)	1.62	0.51	1	0.51	0.51
1.625 - 1.875	1F4Z	1.80	0.36	1	0.36	0.36
1.875 - 2.125	1C8S	2.00	0.41	1	0.41	0.41
2.125 - 2.375	1CWQ(B)	2.25	0.78	1	0.78	0.78
2.375 - 2.625	1IW9	2.50	0.39	1	0.39	0.39
2.625 - 2.875				0		
2.875 - 3.125				0		
3.125 - 3.375	1FBK	3.20	0.79	1	0.79	0.79
