# Supplementary figures and images for: Protein Conformational Changes in the Bacteriorhodopsin Photocycle: Comparison of Findings from Electron and X-Ray Crystallographic Analyses
Source: PLoS One. 2009 Jun 2;4(6):e5769. doi: 10.1371/journal.pone.0005769 (PMC2685002; doi:10.1371/journal.pone.0005769)

## RMSD among late intermediate coordinates

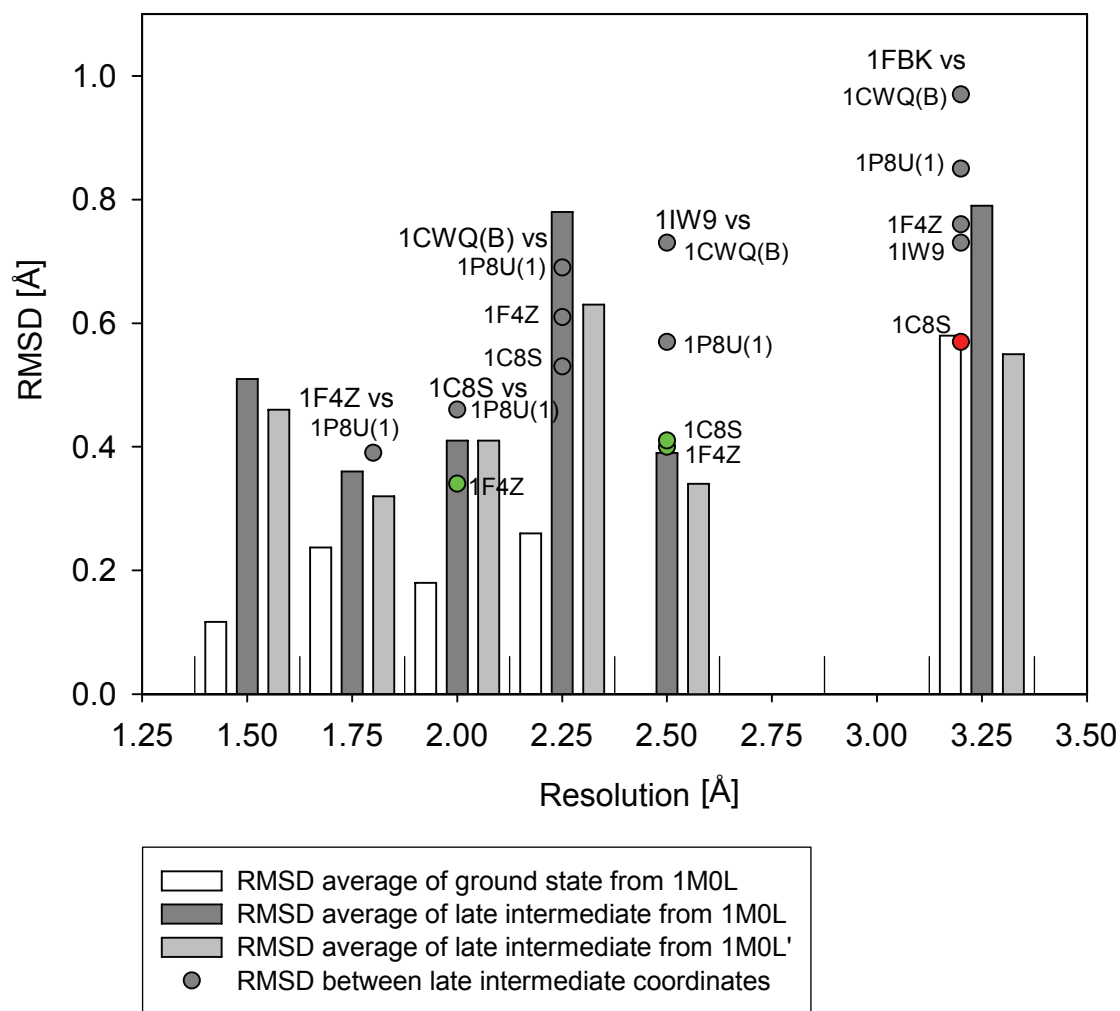

Supplement: Figure S2 — RMSD among late intermediate coordinates. In this figure we calculated the RMSD deviations among late intermediate structures for all possible combinations. RMSD of each possible combination between 6 late intermediate coordinates (M2∼N′, 6×5/2 = 15 cases) is plotted as a circle according to the lower resolution of that pair. Several circles that showed relatively smaller RMSD in that resolution range are colored in red or green and they are discussed later. For comparison RMSD average in each resolution range (0.25 Å interval) was shown as a vertical bar for ground state coordinates or late intermediate coordinates. RMSD values were calculated using the same region where 1M0L is modeled (residues 5–156, 162–231) to ensure that the comparisons were valid across all sets of coordinates. Some coordinate sets, like 1C8S, lack more residues than 1M0L and the RMSD was calculated using the set with the fewer residues. To account for this, the RMSD average of late intermediates from 1M0L was calculated in two ways. The first calculation was conducted simply using the region that overlaps with 1M0L (residues 5–156 and 162–231). The second calculation was conducted using the region where the 1C8S is modeled (residues 5–153 and 176–222) excluding a large area (residues 154–175); this second calculation is represented as 1M0L′ in the figure legend. In principle, the bar graphs of 1M0L′ showed a better RMSD than the bar graph of 1M0L because the latter includes less relatively higher temperature factor regions. Especially the resolution range of 3.25 Å where 1FBK belongs showed a large improvement because the cytoplasmic part of helices E, F, and G of 1FBK significantly deviate from 1M0L. We find that the RMSD distribution between the coordinates of different intermediates is higher than the average RMSD between ground state coordinates suggesting that not all intermediate coordinates represent the same structure. Some combinations have larger RMSD values even when compared to [file pone.0005769.s002.pdf]

30° tilt

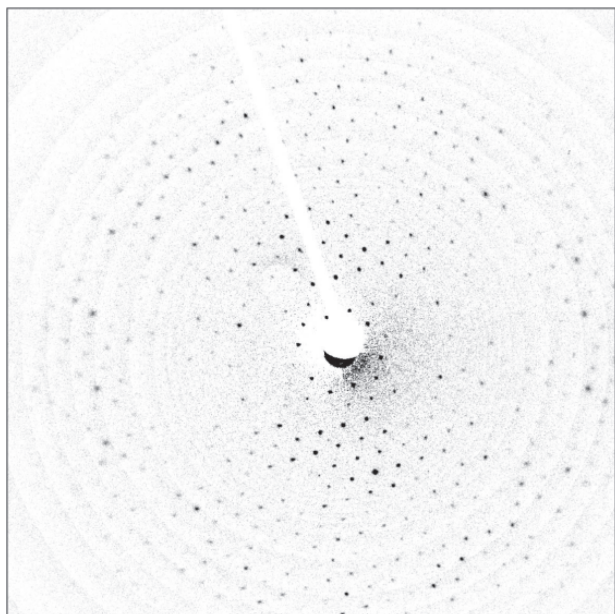

60° tilt

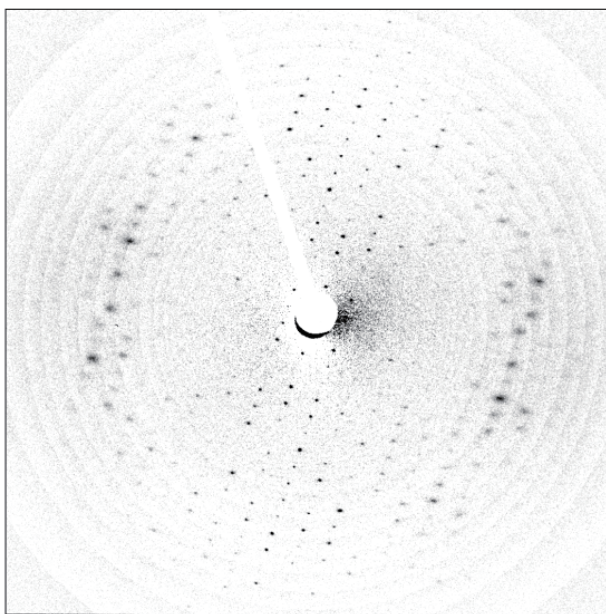

Supplement: Figure S3 — Electron diffraction patterns used to build cytoplasmically open state model. Diffraction patterns were recorded at 120 kV using a CCD camera with 1150×1150 pixels as described before [56]. (a) 30° tilt. (b) 60° tilt. Here we present details of how the electron diffraction patterns were recorded from two-dimensional crystals embedded in a thin film of either glucose or trehalose for determination of the 1FBK structure reported in Subramaniam and Henderson (2000) [27]. The rationale for using sugars such as glucose or trehalose is that we presume they maintain the intrinsic order in the crystal by minimizing its contact with the surface of the carbon film. Untilted diffraction patterns could be recorded to better than 3-Å resolution from most specimens without much difficulty. However, it was considerably more difficult to reproducibly obtain good diffraction patterns at high specimen tilts. At higher tilts, the diffraction patterns are “blurred” in the direction perpendicular to the tilt axis. This is due to vertical distortions (i.e., lack of flatness) introduced in the crystal upon its contact with the carbon film. As a consequence, the angle between the electron beam and the normal to the plane of the crystal is not constant across the entire crystal, and the intensities of spots at higher z* values become spread out over progressively larger areas in the direction perpendicular to the tilt axis. This problem can be partially overcome by recording data from smaller areas of the crystal. Thus, for tilted specimens, diffraction patterns were collected from areas (∼1–1.5 µm2) which were small enough to be sufficiently flat, but large enough to provide a good signal/noise ratio for detection of weak and higher order reflections. There was no clear correlation observed between the qualities of patterns obtained from specimens prepared with either glucose or trehalose; however, it was often the case that in a given session, depending on the age and hydrophobicity of th [file pone.0005769.s003.pdf]

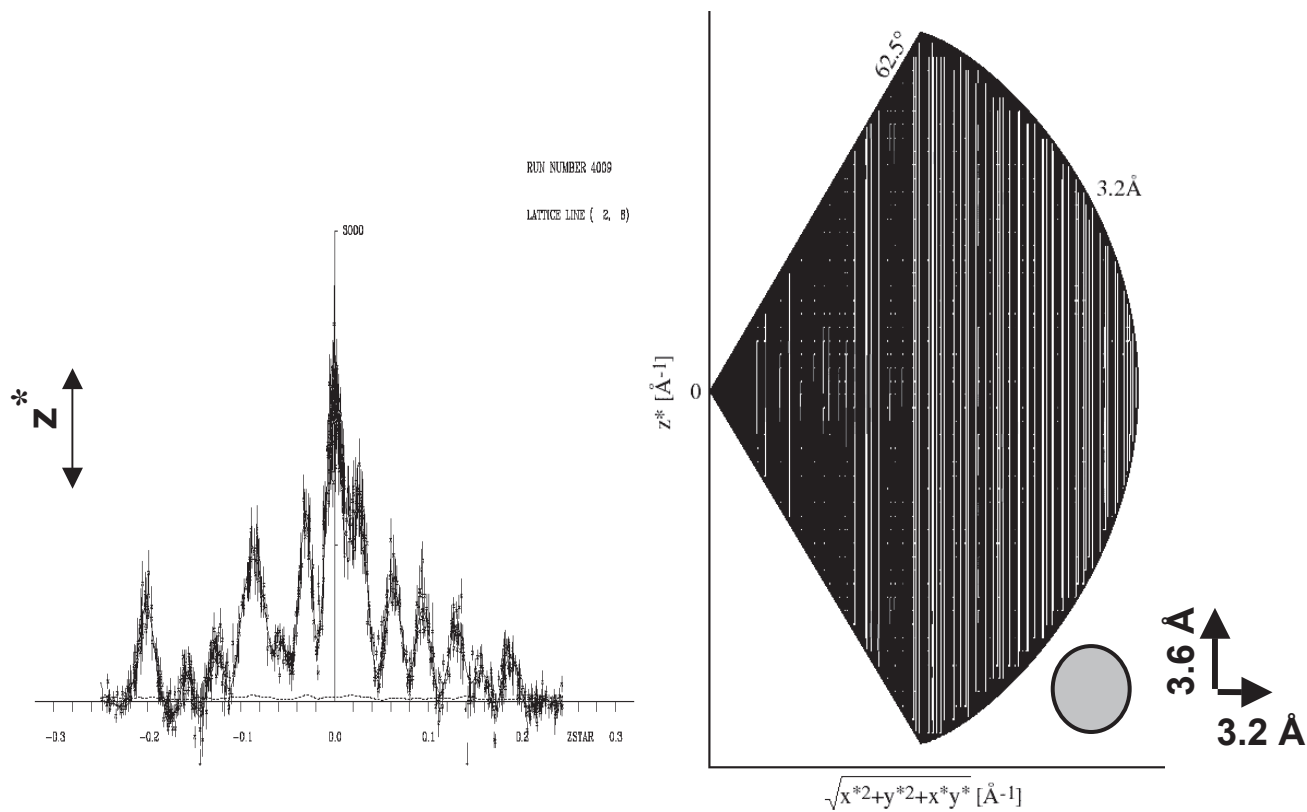

Supplement: Figure S4 — Selected lattice line and the profile of merged set projected to the plain. (A) Selected lattice line, (2,8). (B) The profile of the fully merged set projected to the plain. Point spread function is also shown. Each diffraction pattern was automatically indexed, and the spot intensities were integrated using either a raster (for patterns recorded at specimen tilts less than 30°) or using profile fitting (for patterns recorded at specimen tilts greater than 30°). Each pattern was then compared to the curves recorded for wild-type bacteriorhodopsin in glucose at −100°C [57] and the relative proportions of the four different twins were determined. This exercise was performed with all four theoretically possible relative orientations of the crystal axes with those of the reference curves to ensure that the data were merged correctly. From the initial set of 486 patterns chosen, 286 minimally twinned diffraction patterns were selected in which the major twin proportion was greater than 0.8. These 286 patterns were merged using the wild-type lattice lines as a reference and lattice lines were fitted to the data to obtain an initial approximately merged set of lattice lines describing the structure of the triple mutant. The original set of 486 patterns was then merged against the new lattice curves to redetermine the twin proportions more accurately. The merging parameters for each crystal were inspected carefully again, and 84 crystals for which the major twin proportion was less than 0.70 were excluded from the data set. The remaining 402 untwined diffraction patterns were used to generate a new set of curves using the initial merge set of curves as a reference, and were self-merged to create a further improved set of lattice lines. The nearly merged data were further refined using an improved estimate of sigma values for each reflection, and by the inclusion of an individual weight factor for each diffraction pattern using procedures described by Grigorieff et al [4]. T [file pone.0005769.s004.pdf]

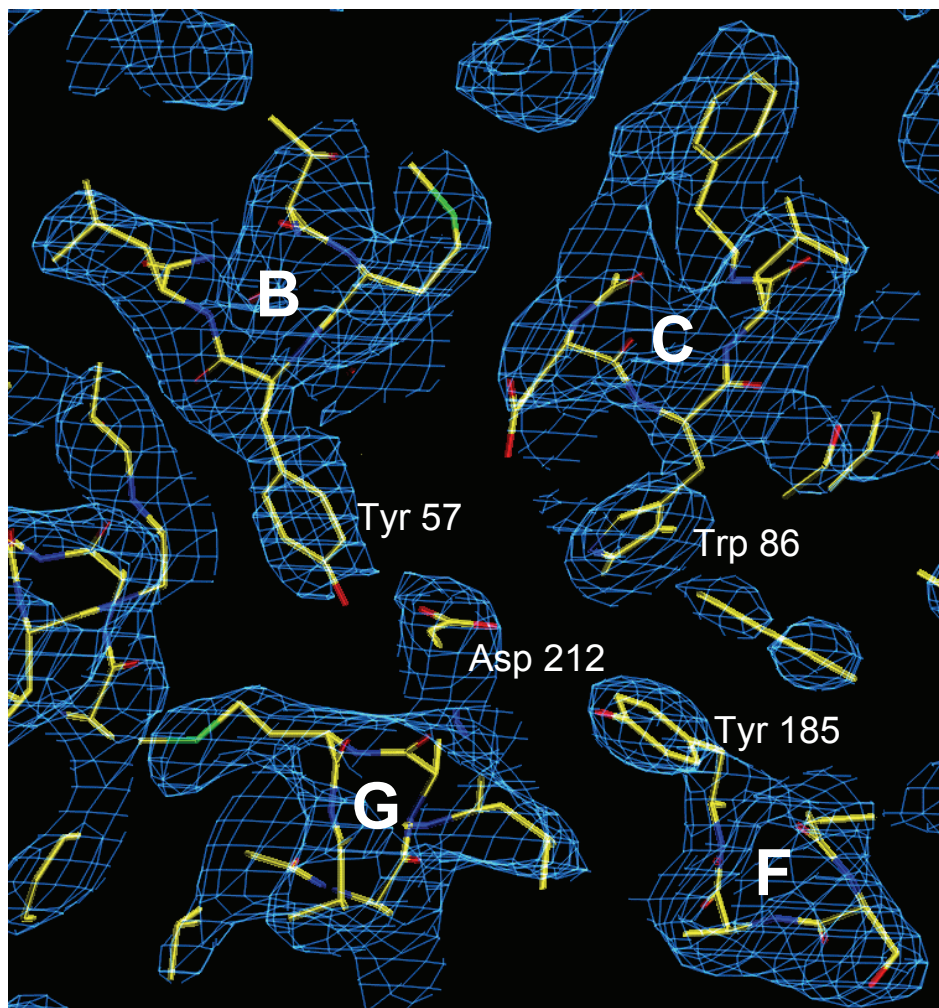

Supplement: Figure S5 — Density (2FO - FC) map of bR triple mutant and refined model (1FBK). In the initial stages of refinement for the previous model, a minimal starting model containing only the transmembrane regions of bacteriorhodopsin was used in a simplified least squares refinement with the PROLSQ program. Coordinates from 6 different starting models (2BRD, 1BRR, 1AP9, 1BRX, 1AT9, and 2AT9) were tested using diffraction data sets obtained both from wild-type bacteriorhodopsin and the triple mutant. We used the diffraction data for refinement of wild-type bacteriorhodopsin reported by Ceska and Henderson [57] as well as those used in the earlier electron crystallographic refinement reported by Grigorieff et al [4]. After a systematic and thorough evaluation, the 1BRR set of coordinates [58] was used as a starting model for the next stage of refinement using the CNS system [59], which involved simulated annealing followed by temperature factor refinement. The validity of the final map was tested by completely omitting from the starting model a series of test residues such as F42, W86, W189, and F208, or various combinations of residues at the cytoplasmic ends of helices F or G. In each case, the difference maps (FO-FC) obtained at the end of the refinement were unambiguous and clear density peaks were observed for each of the omitted regions. (0.94 MB PDF) [file pone.0005769.s005.pdf]
